# A survey of software for genome-wide discovery of differential splicing in RNA-Seq data

**DOI:** 10.1186/1479-7364-8-3

**Published:** 2014-01-21

**Authors:** Joan E Hooper

**Affiliations:** 1 Department of Cell and Developmental Biology, University of Colorado Anschutz Medical Campus, 12801 17th Ave. rm 12103, MS 8108, PO Box 6511, Aurora, CO 80045, USA

**Keywords:** RNA-Seq, Software, Differential splicing, Alternative splicing, Splicing analysis

## Abstract

Alternative splicing is a major contributor to cellular diversity. Therefore the identification and quantification of differentially spliced transcripts in genome-wide transcript analysis is an important consideration. Here, I review the software available for analysis of RNA-Seq data for differential splicing and discuss intrinsic challenges for differential splicing analyses. Three approaches to differential splicing analysis are described, along with their associated software implementations, their strengths, limitations, and caveats. Suggestions for future work include more extensive experimental validation to assess accuracy of the software predictions and consensus formats for outputs that would facilitate visualizations, data exchange, and downstream analyses.

## What is differential splicing analysis and why is it useful?

With the explosion of transcriptome data derived from RNA-Seq, it has become apparent that alternative splicing is a major contributor to cellular diversity in both normal tissues and disease [1-5]. Sets of genes that are regulated by alternative splicing are often different from those that are regulated by differential expression [6] and tend to highlight different biological processes [7,8]. Therefore, differential splicing complements differential gene expression in genomic-level descriptions of gene regulation in biological systems.

Alternative splicing (AS) is prevalent in multicellular organisms, affecting approximately 50%–60% of genes in *Arabidopsis*[9] and approximately 90%–95% of genes in mammals [10,11]. It includes exon skipping, intron inclusion, mutually exclusive exons, and alternative 5′ or 3′ splice sites for an included exon. Along with alternative promoter usage and alternative polyadenylation site usage, AS allows multiple mRNA variants, or isoforms, to be produced by a single gene [10-18]. AS isoforms generate regulatory and functional diversity, differing in untranslated regions that regulate transcript localization, stability, or translation, or in regions encoding protein-protein interactions or sites for post-translational modification [19-21]. AS analysis describes the alternative use of exons and splice sites within a single gene or sample. AS databases, which are listed at [22], and software for AS analysis, which is reviewed elsewhere (e.g., [23]), are beyond the scope of this discussion, which is focused on differential splicing.

Differential splicing analysis describes the differences in AS site usage between two samples. This is critical for studies involving mechanisms of AS and its regulation. In addition, differential splicing analysis describes the differences in splice isoforms between two samples, uncovering functional diversity that is missed by differential gene expression analysis. Available software tools and packages take conceptually different approaches that identify differential splicing at the level of the gene, the exon, or both. The choice of which approach and software is the best to use for a given analysis depends on the experimental objectives and expected outcomes. Table 1 summarizes the software under review, highlighting the strengths of each, their limitations, and directions for future improvements.

**Table 1:** Summary of differential splicing analysis software

	**Model**	**Precision**	**Sensitivity**	**Pair end**	**Multicores**	**Novel events**	**Visualization**	**User-friendly**	**URL**	**Ref**
Cuffdiff 2	Isoform	?	?	☺	☺	☺	☺	☺	[24]	[25]
MISO	Isoform	?	?	☺	☺	☹	☺	☺	[26]	[27]
DEXSeq	Exon	?	?	☹	☺	☹	☺	☹	[28]	[29]
DSGseq	Exon	?	?	☹	☹	☹	☹	☹	[30]	[31]
MATS	Junction + exon	☺☺	?	☺	☹	☺	☹	☺	[32]	[33]
DiffSplice	Junction + exon	?	?	☹	☹	☺	☺☺	☺	[34]	[35]
Splicing compass	Gene	?	?	☹	☺	☹	~	~	[36]	[37]
AltAnalyze (microarray)	Junction + exon	☺	☺☺	☹	☺	☹	☺	☺	[38]	[39]

Excellent (☺☺), very good (☺), good (~), could be improved (☹), unknown (?), multi-cores (supports use of multiple cores to speed computations).

## The challenges for differential alternative splicing analysis

Splicing analysis requires more input data than gene expression analysis because a given gene often has several splice isoforms. A sequencing read can map anywhere along a gene and count towards expression, but a read must include the AS region to count towards splicing analysis. In a recent human study, 100- to 150-M reads (100 nt, paired end) were needed to detect 80% of AS events, and >400-M reads were needed to detect 80% of AS differences between two conditions [40]. Given current sequencing costs and computational approaches, statistically robust detection of differential splicing is biased towards the more abundant transcripts.

The problem is compounded because many genes have multiple AS choices, and each resulting isoform may have biologically unique properties. To reconstruct which full-length isoforms are present in what amounts requires either full-length sequencing of transcripts or full knowledge of all possible isoforms as well as considerable modeling based on the shorter reads. In the future, when high-throughput full-length transcript sequencing becomes cost-effective, splicing analysis will undoubtedly be based on comparison of isoforms. However, most current approaches ignore isoform reconstruction in favor of local analysis.

The challenges associated with differential splicing analysis and the following discussion of differential splicing analysis software apply equally to mammalian systems and to organisms with more compact genomes. The primary difference is that better performance is expected for all approaches in compact genomes, due to their lower complexity [41].

## Three approaches to differential splicing analysis—what does each do?

If the experimental objective is to identify the relative abundance of the encoded alternative protein products, along with linked regulatory information (alternative promoters, alternative 3′ untranslated regions (UTRs)), then isoform modeling with either Cuffdiff 2 [25] or MISO [27] is most appropriate. Here, all reads that have been mapped onto a gene are distributed between its isoforms, producing a probabilistic model of the frequency of each isoform in the original sample. While both MISO and Cuffdiff 2 rely on annotated files of splice isoforms, Cuffdiff can also incorporate novel splicing events that it discovers in the mapped sequencing files. Cuffdiff 2 returns a list of differentially spliced genes, with associated *p* values and false discovery corrections, but with no indication of the exons/junctions involved. Many genes are filtered out due to low coverage and/or wide confidence limits of isoform predictions. MISO uses different statistics and less filtering to return a list of differentially spliced genes. It includes detailed information on the detected splicing differences such as which exon/junctions are involved, alternative splice type (skipped exon, mutually exclusive exons, retained intron, alternative 5′ splice site, and alternative 3′ splice site), magnitude of difference, and coverage. One drawback of MISO is that it lacks statistical methods for handling groups of samples. Thus, MISO gives richer information in two-way comparisons, while Cuffdiff 2 incorporates the added statistical power of multiple samples and/or biological replicates.

For both MISO and Cuffdiff 2, genes with many isoforms present a statistical problem. As the number of isoforms increases, so do the degrees of freedom in the model, such that the confidence intervals for each isoform also increase. Based on this theoretical consideration, isoform reconstruction methods are expected to be biased against detection of differential splicing in genes with many isoforms.

If the experimental objective is more local, dealing with inclusion of specific exons or splice sites, then several choices are available. DEXSeq [23,29] and DSGseq [22,31] focus on exon usage; they equate differential usage of non-terminal exons with differential splicing. Read densities are calculated for every annotated exon in the genome, data dispersion is modeled, and junction information is ignored. Neither will do a simple two-sample comparison; they both require groups of samples and look for differences between groups. They calculate the probability that usage of each exon is different by comparing read densities across the gene between sample groups. DEXSeq reports *p* values for differential usage of each exon, with corrections for false discovery and with associated fold change. DSGseq reports differential exon usage, expressed by a novel NB statistic, for each exon and for each gene, and includes the mean read density across the gene (coverage). DSGseq reports no quantification (e.g., fold change) of the differential exon usages.

The exon-centric approach of DEXSeq and DSGseq is immune to isoform complexity. It is expected to be biased against small exons; this includes alternate 5′ or 3′ splice sites, which are defined as separate exons. Small exons will have lower read counts than large exons, and thus more variability in the relative read densities that are used to calculate differential usage. In addition, the normalization across a gene that corrects for differences in gene expression levels between samples can introduce artifacts. For instance, when a gene has alternative sites for transcription initiation or polyadenylation that generates isoforms with substantially different lengths, and those isoforms are also differentially expressed, these events can be misidentified as differential splicing.

The performance of DEXSeq and DSGseq is heavily dependent on the annotation files, as they only consider annotated exons. RefSeq annotations [42] are conservative and exclude many exons and alternative splices that are likely to be of interest. ENSEMBL and GENCODE (the human ENSEMBL genebuild) annotations [43,44] not only include many more exons and alternative splices, but also include gene fusions (read-through from one gene to the next) that confound normalization for gene expression levels. Thus, analyses based on RefSeq annotations will miss many ‘interesting’ events, while those based on ENSEMBL will lose statistical significance through extensive multiple testing corrections. Therefore, the annotation used should be selected depending on experimental objectives, and it might be prudent to run parallel analyses with each annotation file.

SplicingCompass [25,37] shows great promise as a refinement of DEXSeq and DSGseq. It includes junction information that improves performance on small exons and can accommodate novel (un-annotated) splicing. It detects differential splicing on the gene level instead of considering exons individually. This better accounts for combined effects and reduces the number of statistical tests, thereby reducing the need for multiple testing corrections. On the other hand, it returns information only at the gene level and provides no insight into either the isoforms (with encoded protein products) or which exons or splices are involved. Future releases incorporating these features are very welcome.

MATS [33] takes the most local view, calculating exon inclusion (percent spliced in (PSI)) from junction and exon reads for the exon and its two flanking exons. It then compares values between samples or conditions (groups of samples) to return the probability of differential splicing, expressed as PSI. It recognizes the type of alternative splicing (e.g., skipped exon and retained intron) and returns both *p* value and magnitude (*Δ*PSI) for each alternative splice in the dataset. It can perform either two-sample comparisons or look for differences between groups of samples. This approach has high precision (low false-positive rate), at the expense of some sensitivity [33]. It should be unaffected by isoform complexity, especially alternative promoter or 3′-end usage, and should perform equally well on both annotated and novel splice events. However, the use of flanking exons means MATS is blind to events involving the first or last exon, which contains the untranslated regions (UTRs) where most post-transcriptional regulatory sequences are found. The precise description of the type of alternative splicing is especially valuable where the experimental question involves splicing mechanisms, while the under-representation of UTRs is problematic if the experimental question involves post-transcriptional regulation.

DiffSplice [35] takes a related approach based on alternative splicing modules (ASMs), regions where transcript isoforms diverge. The exon and splice junction data for each gene are represented as a splicing graph (splicing map) that is built from sample data without relying on annotated transcriptomes or pre-determined splice patterns. Alternative splicing generates branches in the splicing graph, which are usually local and modular (ASMs). Thus, a given gene may have several ASMs, each involving one or more alternative exons and splice junctions. DiffSplice then utilizes a non-parametric permutation test to identify significant differences in expression at both gene level and ASM level. Thus, it performs equally well on annotated and novel events, as well as splices involving transcript ends, and is unaffected by isoform complexity. Its precision and sensitivity await rigorous evaluation.

## Usage and user interfaces

All of these packages take as input mapped reads (.bam files) and an annotation (.gtf) file. Only AltAnalyze offers a graphical user interface. The rest are run from the command line. MATS and Cuffdiff 2 offer the most streamlined use. Each has a wrapper script that is called with a single command, as well as excellent documentation at their respective websites. They include detailed instructions for use at the command line, clear explanations of the optional parameters, and step-by-step instructions to insure that input data are appropriately formatted. The outputs of these two packages are user-friendly; the .txt tables are well labeled and intuitive. Cuffdiff 2 also offers CummeRbund [25], an R package for downstream analyses and visualizations.

DiffSplice offers a wrapper script that will run the three steps of the analysis, or each step can be run individually. The descriptions of usage are clear, but the discussion of optional parameters and data interpretation are still under construction. The visualization is a major asset of this software package—tracks of read densities and the deduced alternative splices can be uploaded and viewed on the UCSC genome browser.

DEXSeq, DSGseq, and SplicingCompass run in R, so they are relatively slow. Each requires multiple steps: reformatting the input annotation and .bam files, building exon count files, and running the differential analysis. For DSGseq, the documentation and usage at the website is straightforward; it offers no options and outputs a single data Table R file. DEXSeq offers multiple options for customization, but usage must be gleaned from a combination of the documentation available at Bioconductor, a tutorial, and an example. The key output data table is buried amongst all the metadata and supporting calculations. DEXSeq provides excellent visualizations; it builds a directory of .html files, one per gene, that illustrate a reconstruction of alternative splicing across samples and with supporting numbers. The SplicingCompass tutorial gives straightforward instructions for the 44 commands necessary to prepare and run a six-sample analysis. It also includes a minimal visualization tool that will plot the read coverage of all samples and groups across a gene.

MISO offers remarkable versatility and remains user-friendly in spite of the resulting complexity. This is largely due to the excellent documentation at their website. Its use requires three sequential steps: analyzing ‘events’ in each sample, computing PSI for each sample, and computing differences between samples. There are multiple outputs, depending on the analysis options selected, which include a rich variety of details ranging from splicing event type and coverage to PSI and magnitude of differences. It includes two very useful utilities for downstream analysis. Outputs can be filtered based on their coverage and/or magnitude of change. There is also an excellent visualization utility that shows the modeling and statistical results alongside the raw RNA-Seq data as it maps across exons and junctions. This combination of versatility, ease of use, detailed outputs, and downstream utilities makes MISO a very attractive package.

## Limiting factors—sensitivity, accuracy, and validation

All of these approach methods are limited by sensitivity, a weakness that is common to many or all RNA-Seq-based analyses [40,41]. At commonly used data depths (e.g., 50- to 100-M reads, 50–100 nts), RNA-Seq analyses perform well on highly expressed and thoroughly annotated genes, but less well on genes at moderate or low expression levels and/or with incomplete annotations [41]. The problem with sensitivity can be partially overcome by increasing read depth or read length [40], especially when excellent annotations are available to help distinguish ‘signal’ from ‘noise’ [41,45]. One alternative might be to develop a secondary analysis based on pooling data from samples or conditions that show high correlation in the primary analysis, thereby increasing effective read depth and sensitivity. Another possibility might be to make more effective the use of the information in paired ends to infer exon usage between the paired mapped reads.

Exon junction microarrays (Affymetrix HJAY or MJAY arrays, Santa Clara, CA, USA [46]) offer an oft-overlooked approach with higher sensitivity than RNA-Seq for well-annotated alternative splices. A software package that integrated RNA-Seq and Exon junction array data would be invaluable, where the experimental objective is a comprehensive description of splicing and splicing differences. AltAnalyze [39], which was designed to process splice-sensitive array data, is a unified and flexible package that can process raw microarray data (or RNA-Seq data in .bed format), compute differences in alternative splicing (exon and junction usage), and integrate with a multitude of other data types (e.g., protein domains and microRNA binding annotations). It offers an interactive graphical user interface that allows users with little knowledge of bioinformatics programs or scripting to customize analyses for their data and to visualize outputs in Cytoscape or other useful formats. While AltAnalyze can be applied to RNA-Seq data, it only analyzes known alternative splicing, which limits its attractiveness in RNA-Seq applications.

For all of these analysis tools and packages, the goal is accuracy, which is limiting false-positives while maximizing true-positives. Accuracy is best determined by extensive experimental validation, for instance, by using quantitative reverse transcribed-polymerase chain reaction (qRT-PCR), to measure the ratios of alternative splice products in the same RNA samples that were used for RNA-Seq (or microarray probing). High-throughput qRT-PCR technologies allow validation at genomic scale (e.g., [5]). MATS used qRT-PCR to validate 164 detected alternative splices that covered the range of false discovery rates [33]. They found that their calculated false discovery rate (fdr) predicted validation rates; for example, splices with fdr < 0.2 had >80% validation rate. SplicingCompass showed promise for accuracy in extensive *in silico* analyses [37], but it remains to be experimentally tested. The global accuracy of DEXSeq, DSGseq, DiffSplice, and Cuffdiff 2 predictions has yet to be experimentally tested. Given the complementary nature of the various approaches described above, it is recommended to try more than one software tool on a dataset and then compare and/or amalgamate the outputs.

This survey has been cursory, and other good splicing analysis software are available [23]. For instance, GLiMMPS is a newly developed statistical method for detecting splicing quantitative trait loci (sQTLs) from RNA-Seq data [47]. Two key goals downstream of alternative splicing analysis are to identify the regulatory differences in the transcript isoforms and the functional differences in the encoded protein isoforms. In this respect, ASPicDB (http://www.caspur.it/ASPicDB/) is a database of human protein variants generated by AS [48]. AltAnalyze has begun to address the consequences of AS by incorporating protein domain annotations into its integrated analysis. A useful development would be standardized format(s) for outputs of differential splicing analyses, like the .bam or .bed formats for genomic information. This would facilitate development of downstream modules that could be applied to any differential splicing analysis for further exploration of functional consequences of variations in splicing.

## Abbreviations

AS: Alternative splicing; ASMs: Alternative splicing modules; fdr: False discovery rate; PSI: Percent spliced in; qRT-PCR: Quantitative reverse transcribed-polymerase chain reaction; UTRs: Untranslated regions.

## Competing interests

The author declares that she has no competing interests.

## Authors' information

JH is trained in and built a career as an experimentalist in developmental biology, molecular genetics, and signal transduction. She has recently begun to employ genomic and bioinformatic approaches to developmental biology. She approaches the topic of software as an experienced biologist and a bioinformatics novice.
